# Tianma Gouteng decoction for essential hypertension: Protocol for a systematic review and meta-analysis

**DOI:** 10.1097/MD.0000000000009972

**Published:** 2018-02-23

**Authors:** Benjian Chen, Yuanping Wang, Zhuogen He, Dawei Wang, Xia Yan, Pingchang Xie

**Affiliations:** a The Second Affiliated Hospital of Guangzhou University of Chinese Medicine, Guangdong Provincial Hospital of Chinese Medicine; b The Second Clinical College of Guangzhou University of Chinese Medicine, Guangzhou, China.

**Keywords:** essential hypertension, protocol, systematic review, Tianma Gouteng decoction

## Abstract

**Background:**

Essential hypertension is one of the most common chronic diseases, as well as one of the leading risk factors for cardio-cerebrovascular diseases. Tianma Gouteng decoction has been commonly used in clinical practice for patients with essential hypertension. It is important to update the search and evaluation to provide the best available evidence for essential hypertension. Here, we provide a protocol to evaluate the efficacy and safety of Tianma Gouteng decoction (TGD) for essential hypertension.

**Methods:**

Retrieve literature on the TGD treatment for essential hypertension in the databases including PubMed, Cochrane Library, EMBASE, Chinese Biomedical database (CBM), Chinese National Knowledge Infrastructure (CNKI), Wanfang database, and Chinese Science and Technology Periodical database (VIP) on computer. The literature to be collected will be those published from the time when the respective databases were established to January 2018. The therapeutic effects according to the blood pressure (BP) will be accepted as the primary outcomes. We will use RevMan V.5.3 software as well to compute the data synthesis carefully when a meta-analysis is allowed.

**Results:**

This study will provide a high-quality synthesis of current evidence of TGD for essential hypertension from several aspects including BP, life quality, heart rate, and adverse events.

**Conclusion:**

The conclusion of our systematic review will provide evidence to judge whether TGD is an effective intervention for patient with essential hypertension.

**PROSPERO registration number:**

PROSPERO CRD 42018082319.

## Abstract 2

Supplemental Digital Content is available in the text

## 1 Introduction

Essential hypertension is defined as high blood pressure that no secondary cause has been found and the patients diagnosed with the disease have systolic pressure >140 mmHg, or diastolic pressure >90 mmHg.^[1]^ Hypertension is one of the most common chronic diseases, as well as one of the leading risk factors for cardio-cerebrovascular diseases.^[2]^ Their major complications include stroke, myocardial infarction, heart failure, and chronic kidney disease, which not only result in high disability and fatality rate but need great healthcare and social resources input, a heavy burden for both the families and national healthcare system.^[3,4]^ It is estimated that 1 billion people have high blood pressure worldwide, and about 7.1 million deaths are linked to the disease every year.^[5]^ High blood pressure is becoming increasingly common due to prolonged life expectancy, widespread obesity, reduced physical activity, and unhealthy diets.^[6]^

Complementary and alternative medicine (CAM) is the term for health therapies and medical products that have typically not been part of conventional medicine and treatments.^[7]^ They may lack a medical explanation and support, however, part of CAM therapies (physical therapy, diet, acupuncture, and traditional Chinese medicine [TCM]) are widely accepted.^[8]^ Today, more patients with cardiovascular disease adopt CAM treatment. TCM is one of the most important parts of CAM, and many studies have shown that Chinese herbal medicine or acupuncture may help control blood pressure.^[9–11]^

Tianma Gouteng decoction (TGD), a TCM compound, is composed of 11 commonly used herbs (Uncaria, *Gastrodia elata*, *Scutellaria baicalensis* Georgi, *Eucommia ulmoides* Oliv, achyranthes root, *Loranthus parasiticus*, abalone shell, Gardenia, *Leonurus japonicus*, caulis polygoni multiflori, and Poria cocos), all of which are standardly recorded in Chinese Pharmacopoeia 2015 edition. It has been used widely in recent decades in China to clinically treat symptoms caused by high blood pressure, such as dizziness and headache, etc.^[12]^ A new animal study shows that TGD could alleviate cognitive dysfunction in rats with hypertension, which was possibly by inducing alteration of glucose metabolism in different brain regions with corresponding functions.^[13]^

Although only 1 systematic review^[12]^ on the TGD treatment for essential hypertension was published 5 years ago, there has been many high quality randomized controlled clinical studies published^[14,15]^ in recent years. Therefore, it is important to update the search and evaluation to provide the best available evidence for essential hypertension. Our systematic review and meta-analysis will answer 2 clinical questions about the TGD treatment for the disease: whether TGD is significantly more effective and safer than the traditional antihypertensive drugs or placeboes; whether the conventional blood pressure medication assisted by TGD is more effective and safer than using the conventional drugs alone.

## 2 Methods

### 2.1 Inclusion criteria for study selection

#### 2.1.1 Types of studies

All randomized controlled trials (RCTs) in both Chinese and English will be included in the research, regardless of whether blinding or allocation concealment was adopted.

#### 2.1.2 Types of patients

The objects of the study will be patients clinically diagnosed with essential hypertension, who have systolic pressure >140 mmHg and/or diastolic pressure >90 mmHg or take antihypertensive drugs.

#### 2.1.3 Types of interventions

The experimental group used TGD or combined with other conventional antihypertensive drugs, the control group of drugs for conventional antihypertensive drugs or placebo.

#### 2.1.4 Types of outcome measures

##### 2.1.4.1 Primary outcomes

The primary outcome measure was blood pressure.

##### 2.1.4.2 Secondary outcomes

- Life quality
- Heart rate
- Adverse events

### 2.2 Search methods for the identification of studies

#### 2.2.1 Electronic searches

Retrieve literature on the TGD treatment for essential hypertension in the databases including PubMed, Cochrane Library, EMBASE, Chinese Biomedical database (CBM), Chinese National Knowledge Infrastructure (CNKI), Wanfang database, and Chinese Science and Technology Periodical database (VIP) on computer. The literature to be collected will be those published from the time when the respective databases were established to January 2018. All references that can meet the inclusion requirements of the study will be reviewed one by one to avoid omission. TGD and essential hypertension will be the search terms. The search strategy of PubMed will be shown in Appendix A as an example, and similar strategies will be applied to other databases.

#### 2.2.2 Searching other resources

In the meantime, the relevant studies published on journals, along with their references, will be manually reviewed and retrieved as the supplementary literature.

### 2.3 Data collection and analysis

#### 2.3.1 Selection of studies

**Figure 1:**
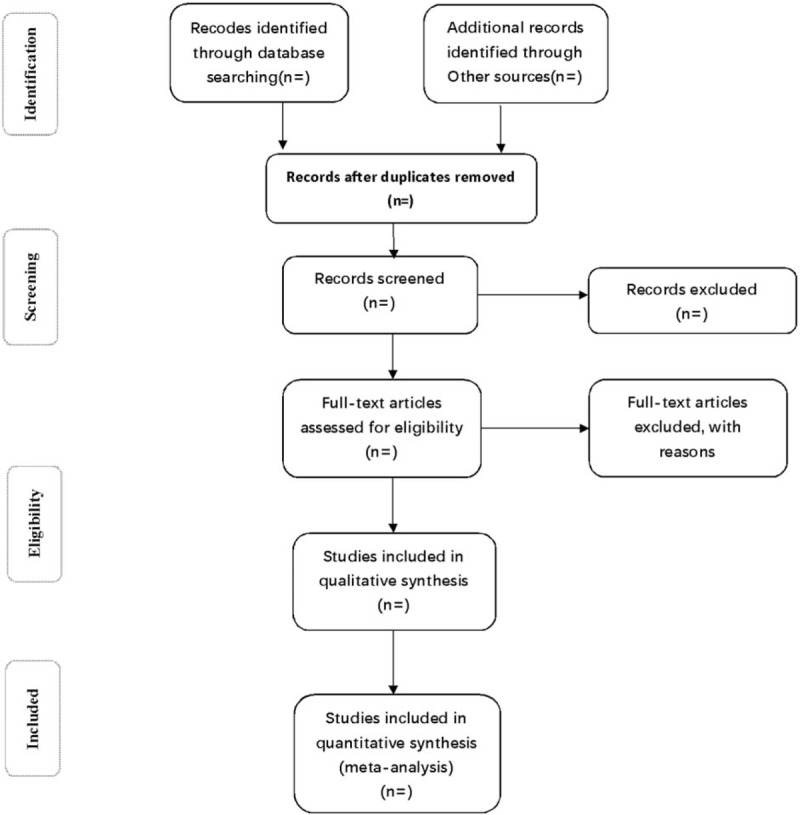
Flow diagram of study selection process.

Two researchers will independently conduct and cross-check the literature screening. In case of disagreement, they shall discuss with each other to come up with a solution or consult another research member. As for the information unavailable, the reviewers shall contact the author of the original paper. They will read the titles of the references first to exclude the literature that are obviously irrelevant, and then read the abstract and full text to determine whether the studies would be finally included. The process of studies selection is presented in a Preferred Reporting Items for Systematic Review and Meta-analysis (PRISMA) flow diagram (Fig. 1).

#### 2.3.2 Data collection and management

Two researchers will retrieve the following data independently according to the data collection table designed beforehand: information about the included trials, such as research topic, first author, which journal and when it was published etc., research design and key elements of quality evaluation, information about the patients in the treatment and control group, including how many cases are included and the ages of patients, etc., outcome indicators, relevant indicators of bias risk assessment.

#### 2.3.3 Assessment of risk of bias in included studies

Two evaluators will assess the risks of the included literature using the tool provided by Cochrane Handbook for Systematic Reviews of Interventions V.5.1.0. The tool includes 7 parts, that is, random sequence generation, allocation concealment, blinding method for patients, researchers and outcomes assessors, incomplete result data, and selective reports. The evaluation results will be divided into 3 levels, namely, low-risk, unclear, and high-risk. They will cross-check the evaluation and data retrieval. Where there are inconsistencies, the problem will be resolved through group discussion, or decided by another researcher.

#### 2.3.4 Measures of treatment effect

The study will employ RevMan 5.3 (Version 5.3, Copenhagen: The Nordic Cochrane Center, The Cochrane Collaboration, 2014) to conduct meta-analysis. The rate ratio will be used to indicate the enumeration data while the mean difference (MD) the measurement data. A 95% confidence interval (CI) will be adopted to present the effect sizes.

#### 2.3.5 Dealing with missing data

If the required data are not clear or not reported in clinical papers, the reviewers will collect the missing data with the data collection table by contacting the first or corresponding author of the studies via phone, e-mail, or mail.

#### 2.3.6 Assessment of heterogeneity

The heterogeneity of the results of the included studies will be analyzed through chi-squared test (*α* = 0.1), with its value determined by *I*^2^. The heterogeneity among the trials will be considered significant if *I*^2^ is >50%, and further subgroup analysis will be carried out to investigate the potential causes.

#### 2.3.7 Assessment of reporting bias

The visual asymmetry on a funnel plot will be used to judge whether a publication bias exists if at least 10 trials are included in the study.

#### 2.3.8 Data synthesis

The meta-analysis will be performed using the fixed effects model if no statistic heterogeneity among the results of the study is found. On the other hand, the random effect model will be employed to carry out the analysis with the effect of the obvious clinical heterogeneity excluded if there is a statistical heterogeneity. When obvious clinical heterogeneity is observed, the researchers can turn to the subgroup or sensitivity analysis, or only descriptive analysis. *α *= 0.05 is used to evaluate the meta-analysis.

#### 2.3.9 Subgroup analysis

Subgroup analysis will be performed based on different interventions, controls, and outcome measures. Adverse effects will be assessed and tabulated with descriptive techniques.

#### 2.3.10 Sensitivity analysis

If *P* value of the heterogeneity is <.1 when the data extraction is examined, and subgroup analysis performed, the meta-analysis will be reconducted with the low-quality studies ruled out.

#### 2.3.11 Ethics and dissemination

The outcomes of this systematic review will offer implications of the use of TGD treatment for essential hypertension patients. It uses aggregated published data instead of individual patient data and does not require an ethical board review and approval. The findings will be published in a peer-reviewed journal and disseminated in conference presentations. It will provide the latest analysis of the currently available evidence for TGD treating essential hypertension.

## 3 Discussion

**Figure 2:**
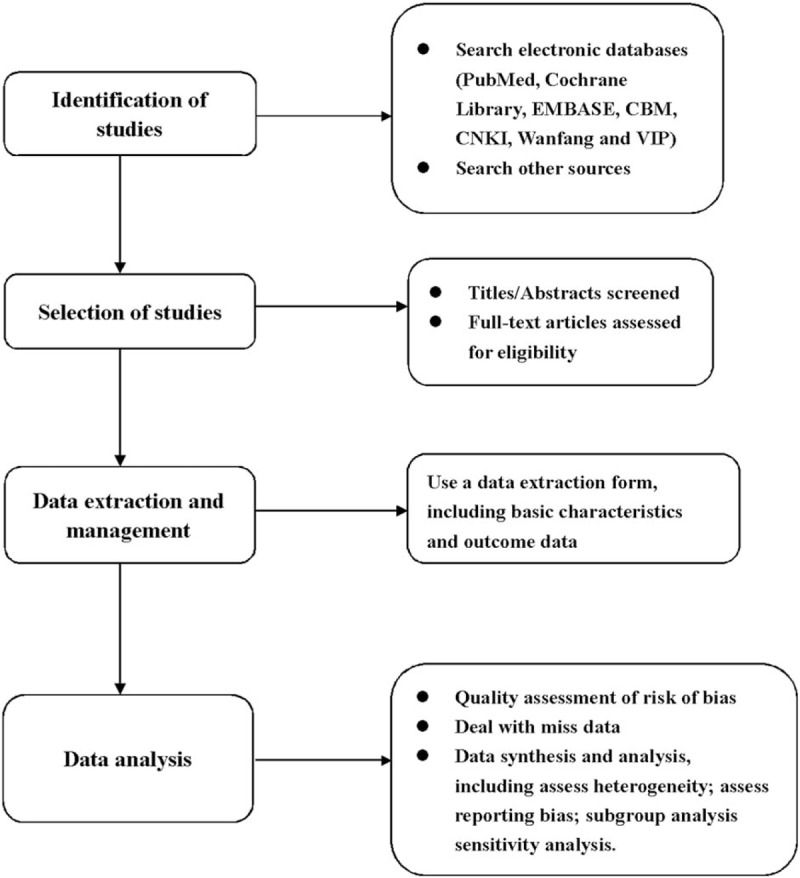
Flow diagram of the systematic review and meta-analysis.

Currently, the first-line drugs used in the treatment of essential hypertension include thiazide diuretics, long-acting calcium channel blockers, angiotensin-converting enzyme inhibitors (ACEI), and angiotensin II receptor blockers (ARBs).^[16]^ However, long-term use of these drugs can bring certain side effects to patients, such as headache, facial flush, and other symptoms. TGD may be an alternative to effectively treat essential hypertension with less side effects. Whereas the efficacy and safety of TGD in treating essential hypertension are uncertain. Therefore, it is necessary to conduct a high quality systematic review and meta-analysis, in which our rigorous approach will provide objective evidence for TGD treatment for essential hypertension. The process of performing this systematic review, shown in Fig. 2, will be separated into 4 parts: identification of studies, selection of studies, data extraction and management, and data analysis. Nevertheless, the study may have some limitations. First, only the literature in Chinese and English will be searched, which may lead to language bias. Second, the different doses of TGD adopted in the interventions of the included studies may lead to significant heterogeneity.

Preferred Reporting Items for Systematic review and Meta-Analysis Protocols (PRISMA-P) checklist of this protocol is presented in online supplementary.

## Supplementary Material

Supplemental Digital Content
