# Supplementary material for: Tianma Gouteng decoction for essential hypertension: Protocol for a systematic review and meta-analysis
Source: Medicine (Baltimore). 2018 Feb 23;97(8):e9972. doi: 10.1097/MD.0000000000009972 (PMC5841972; doi:10.1097/MD.0000000000009972)
Supplement: Supplemental Digital Content [file medi-97-e9972-s001.docx]

**Appendix A.**

***Search strategy used in PubMed database***

#1 [essential hypertension](https://www.ncbi.nlm.nih.gov/mesh/2023415)

#2 tianma gouteng decoction OR tian ma gou teng decoction OR tianma gouteng tang OR tianma gouteng yin

#3 Randomized controlled trial OR clinical study OR Clin-ical Trial OR Controlled study OR Controlled Trial OR Random*Control* study OR random* Control* Trial

#1 AND #2 AND #3
